# Complete Genome Sequences of Four Bovine Coronavirus Isolates from Pennsylvania

**DOI:** 10.1128/genomeA.00467-18

**Published:** 2018-05-31

**Authors:** Maurice Byukusenge, Ruth Helmus Nissly, Sunitha Manjari Kasibhatla, Lingling Li, Rebekah Russell, Hayley Springer, Rhiannon Barry, Robert Van Saun, David Wolfgang, Ernest Hovingh, Urmila Kulkarni-Kale, Suresh V. Kuchipudi

**Affiliations:** a Animal Diagnostic Laboratory, Department of Veterinary and Biomedical Sciences, The Pennsylvania State University, University Park, Pennsylvania, USA; b Department of Veterinary and Biomedical Sciences, The Pennsylvania State University, University Park, Pennsylvania, USA; c Bioinformatics Centre, INDI, Pune, India; d HPC-Medical & Bioinformatics Group, Centre for Development of Advanced Computing, Savitribai Phule Pune University, Pune, India

## Abstract

We report four full-genome sequences of bovine coronavirus (BCoV) isolates from dairy calves in Pennsylvania obtained in 2016 and 2017. BCoV is a pathogen of great importance to cattle health, and this is the first report of full-genome sequences of BCoV from PA cattle.

## GENOME ANNOUNCEMENT

Bovine coronavirus (BCoV), a member of the Betacoronavirus genus of the Coronaviridae family, is an enveloped virus with an approximately 31-kb single-stranded positive-sense RNA genome. Infection of cattle with bovine coronavirus is a major contributor to diarrhea in calves, is involved in the etiology of winter dysentery in adult cattle, and serves as a contributing pathogen in bovine respiratory disease complex (1, 2).

Coronaviruses are difficult to cultivate in the laboratory, and only 17 full-genome sequences of unique BCoV isolates are publicly available in GenBank (3). We generated full-genome sequences of four BCoV isolates, 7-16-23, 4-17-03, 4-17-08, and 4-17-25, recovered from fecal samples of 3- to 14-day-old dairy calves in July 2016 and April 2017. Fecal samples were screened for presence of BCoV genetic material by real-time reverse transcription-PCR (qRT-PCR), as described previously (4). The study has been approved by the Pennsylvania State University Institutional Animal Care and Use Committee (IACUC protocol no. 46948). Fecal extracts from positive samples were cultured on HRT-18G cells, and after 3 to 5 days, culture medium samples were rescreened by qRT-PCR. Libraries were prepared from viral RNA extracted from positive-culture media using the TruSeq stranded mRNA kit without the poly(A) selection step. Genetic material was subjected to whole-genome sequencing using a 150-nucleotide (nt) read-length single-read approach on the Illumina MiSeq platform. Sequence files were assembled with the BCoV Mebus strain (GenBank accession number U00735) as a reference using SeqMan NGen build 15.0.1.1.

The four sequences generated contained 31,016 to 31,032 nucleotides, with 99.36% nucleotide identity between isolates. Following alignment of full-genome nucleotide sequences by the MUSCLE algorithm implemented in the MEGA7 package (5, 6), phylogenetic analysis indicated that the four isolates were closely related to one another and shared 99% coverage and 99% identity with their closest relative, sable antelope coronavirus US/OH1/2003 (GenBank accession number EF424621). Other closely related sequences were those of isolates from diverse cattle and captive ruminants obtained in Ohio, a neighboring state with a border approximately 270 km away from the collection sites of these samples.

The ORF1ab, HE, S, NS5, E, M, and N genes of the four sequences were subjected to BLASTN analysis (7, 8). According to these analyses, the ORF1ab and HE genes of the four isolates were closely related to the strain calf-giraffe coronavirus US/OH3/2006 (GenBank accession number EF424624), with >99% nucleotide identity. Similarly, the S, E, and M genes from all four isolates had >99% nucleotide identity with sequences with GenBank accession numbers HE616738 (strain VB 7/09/MAYABEQUE/2009), KX982264 (strain BCoV_2014_13), and EF424615 (strain E-AH65), respectively. Of interest, the first codon position of the E gene of 4-17-25 contained a point mutation shifting ATG to ACG. The N gene sequence was similar to that of BCoV strain E-AH65 in most of the isolates, but that of 4-17-08 shared >99% nucleotide identity with that of the giraffe coronavirus US/OH3/2003 strain (GenBank accession number EF424623).

### Accession number(s).

The complete genome sequences of the BCoV isolates 4-17-03, 4-17-25, 4-17-08, and 7-16-23 have been deposited in GenBank under the accession numbers MH043952 to MH043955.
