# Erratum for Byukusenge et al., “Complete Genome Sequences of Four Bovine Coronavirus Isolates from Pennsylvania”

**DOI:** 10.1128/MRA.00845-18

**Published:** 2018-07-19

**Authors:** Maurice Byukusenge, Ruth Helmus Nissly, Sunitha Manjari Kasibhatla, Lingling Li, Rebekah Russell, Hayley Springer, Rhiannon Barry, Robert Van Saun, David Wolfgang, Ernest Hovingh, Urmila Kulkarni-Kale, Suresh V. Kuchipudi

**Affiliations:** a Animal Diagnostic Laboratory, Department of Veterinary and Biomedical Sciences, The Pennsylvania State University, University Park, Pennsylvania, USA; b Department of Veterinary and Biomedical Sciences, The Pennsylvania State University, University Park, Pennsylvania, USA; c Bioinformatics Centre, Savitribai Phule Pune University, Pune, India; d HPC-Medical & Bioinformatics Group, Centre for Development of Advanced Computing, Savitribai Phule Pune University, Pune, India

## ERRATUM

Volume 6, no. 22, e00467-18, 2018, https://doi.org/10.1128/genomeA.00467-18. Page 1: The author affiliations should appear as given in this erratum.

